# Characteristics and outcomes of emergency patients with self-inflicted injuries: a report from ambulance records in Osaka City, Japan

**DOI:** 10.1186/s13049-016-0261-0

**Published:** 2016-05-11

**Authors:** Tasuku Matsuyama, Tetsuhisa Kitamura, Kosuke Kiyohara, Sumito Hayashida, Takashi Kawamura, Taku Iwami, Bon Ohta

**Affiliations:** Department of Emergency Medicine, Kyoto Prefectural University of Medicine, Kamigyo-ku, Kyoto, 602-0841 Japan; Division of Environmental Medicine and Population Sciences, Department of Social and Environmental Medicine, Graduate School of Medicine, Osaka University, 2-2 Yamadaoka Suita, Osaka, 565-0871 Japan; Department of Public Health, Tokyo Women’s Medical University, 8-1, Kawada-cho, Shinjuku-ku, Tokyo, 162-0054 Japan; Osaka Municipal Fire Department, 1-12-54 Kujo Minami, Nishi-ku, Osaka, 550-8566 Japan; Kyoto University Health Service, Yoshida-Honmachi, Sakyo-ku, Kyoto, 606-8501 Japan

**Keywords:** Self-inflicted injuries, Epidemiology, Population-based study, Emergency medical service

## Abstract

**Background:**

Few studies have evaluated the actual situations of emergency patients with self-inflicted injuries treated by emergency-medical-service (EMS) personnel.

**Methods:**

This study retrospectively reviewed population-based ambulance records in Osaka City, Japan, between January 2010 and December 2012, and enrolled emergency patients who suffered from self-inflicted injuries such as poisoning by drugs or gas, cutting/piercing skin, jumping from heights, hanging, and drowning. The endpoint was the annual incidence per 100,000 populations in Osaka City of emergency patients who presented with self-inflicted injuries by age and sex. Their outcomes including deaths at the scene and hospital arrival were also evaluated.

**Results:**

During the study period, a total of 8,671 patients with 9,424 incidents of self-inflicted injuries were documented. The annual incidence of self-inflicted injuries was higher among women than men in the whole population and in the age group < =49 years (136.9 versus 82.6, and 214.8 versus 93.3, both Ps < 0.001), but it was inversely lower among women in the age group > =50 years (49.0 versus 68.9, *P* < 0.001). The total number of self-inflicted deaths was 1,564 (16.6 %), and the overall proportion of self-inflicted deaths was greater among men than women (32.2 % [1075/3340] vs. 7.5 % [451/6027], *P* < 0.001). The proportion of self-inflicted hanging was 76.7 % [1142/1489], followed by poisoning by carbon monoxide at 57.1 % [56/98] and jumping to death at 47.6 % [254/534].

**Discussion:**

Using large-scale EMS records, we investigated characteristics and outcomes of emergency patients with self-inflicted injuries treated by EMS personnel. Our findings suggested the gender paradox that the proportion of self-inflicted deaths was higher among men than women, while the proportion of non-fatal self-inflicted injuries was higher among women than among men, particularly in the group aged <=49 years. Our findings showing the importance of the prevention for self-inflicted injuries as well as the gender paradox of self-inflicted injuries will provide important epidemiological information to improve psychiatric cares in prehospital emergency settings.

**Conclusions:**

In the total population, the annual incidence of self-inflicted injuries responded to by EMS personnel was higher among women than among men. However, the proportion of self-inflicted deaths was greater among men than women, and the most frequent manner among deceased patients was by hanging.

## Background

Recently, suicide has become a very important public health problem, and its incidence has been increasing worldwide. Therefore, many countries make great efforts to develop prevention strategies for suicide [1]. It is well-known that characteristics of self-inflicted injuries vary exceedingly between countries or ethnicities [1–3]. Organization for Economic Co-operation and Development (OECD) reported that while the average annual incidence of suicide per 100,000 persons in all OECD countries was 12.4, the annual incidence in Japan was 21.4, which was quite high even among the industrialized countries [4]. Thus, developing a prevention strategy for suicide is an urgent issue in Japan.

Self-inflicted injuries include any violence directed against oneself, with or without suicidal intent [5]. The World Health Organization (WHO) estimated that non-fatal self-inflicted injuries might occur 20 times or more frequently than fatal suicidal behavior every year [1]. Non-fatal self-inflicted injuries are often repeated and have the strongest risk factor for future suicide [6–9]. Importantly, most previous studies regarding self-inflicted injuries have collected data based on self-report questionnaires or hospital-based medical records about suicidal behavior, and only a few population-based studies have evaluated the characteristics and outcomes of emergency patients with self-inflicted injuries treated by emergency-medical-service (EMS) personnel [1, 10, 11].

Using the EMS records of the period from 2010 to 2012 in Osaka City, which is the largest metropolitan community in western Japan with approximately 2.7 million inhabitants, we conducted a population-based epidemiological study on emergency patients with self-inflicted injuries treated by EMS personnel. The aim of this study was to assess their characteristics and outcomes and to provide fundamental information for the intervention and prevention of self-inflicted injuries including suicide.

## Methods

### Study design, population, and setting

In this descriptive study, we retrospectively reviewed the EMS records of the Osaka Municipal Fire Department from January 2010 to December 2012. All patients who presented with self-inflicted injuries and were treated by EMS personnel in Osaka City were enrolled in this study. If two or more incidents of self-inflicted injury methods were documented in one patient, we treated each of them separately. Self-inflicted injury events were categorized by the EMS/physicians as follows in accordance with regular forms based on a previous study; poisoning by drugs (‘sleeping pill or tranquilizer’ or ‘other drugs’), poisoning by gas (‘carbon monoxide [CO]’ or ‘other gas’), cutting and/or piercing skin (‘wrist or arm’ or ‘other part of body’), jumping from heights, hanging, and drowning [12]. For emergency patients transported to a hospital, the diagnosis of self-inflicted injuries was made clinically by the physician caring for the patient, in collaboration with EMS personnel. For patients treated at the scene without transportation, the diagnosis was made by EMS personnel based on on-site observations and an EMS interview with the patient. This study was approved by the Ethics Committee of Kyoto University Graduate School of Medicine and the Ethics Committee of Kyoto Prefectural University of Medicine. Personal identifiers were already removed from the database by EMS personnel. The requirement of informed consent of patients was waived.

### EMS system in Osaka City

Osaka City has an area of 222 km^2^, and a population is approximately 2.7 million in 2010 (population density, about 12,000 persons/km^2^). Details of the municipal EMS system in Osaka City were previously described [13]. The system is operated by the Osaka Municipal Fire Department covering entire ambulance service in Osaka City and is activated by dialing the emergency number “119” on a telephone. In 2010, Osaka City had 25 fire stations and one dispatch center (60 ambulances in total). Each ambulance typically operates with a crew of three emergency care providers, including at least one highly trained emergency life-saving technician. Osaka City had 186 hospitals (32,922 beds) in 2012, 94 of which, along with six critical medical care centers, were equipped to treat patients with life-threatening emergencies [14]. Emergency dispatchers in Osaka City did not make phone-calls to hospitals for patient acceptance, leaving it to ambulance crews to select the appropriate hospital, including critical care medical centers, to which to deliver patients requiring emergency care [14].

### Data collection and quality control

The following data were uniformly collected via regular forms including age, sex, location of call, type of self-inflicted injuries, time and date of all events, time-course of transportation, type of hospitals transported to, and their clinical departments, and patient outcomes. The forms were completed by EMS personnel in cooperation with the physicians caring for the patient, transferred to the EMS Information Center of Osaka Municipal Fire Department, and then checked by the investigators. If any data were incorrect, investigators in the EMS information center returned the form to the relevant EMS personnel for completion.

### Endpoints

The endpoint of this study was the annual incidence per 100,000 population in Osaka City of emergency patients with self-inflicted injuries by age and sex. Their outcomes including deaths at the scene and hospital arrival were also evaluated. Patient outcomes were classified as follows: refusal of transport by patients, only prehospital treatments at the scene, no hospital admission after transportation, hospital admission, or death (confirmed at the scene or at hospital arrival).

### Statistical analysis

Incidences and outcomes of self-inflicted injuries were compared considering the personal background characteristics and temporal patterns using either the chi-square test or Fisher’s exact test. Annual incidence per 100,000 population in Osaka City of emergency patients with self-inflicted injuries by age (10-year strata) and sex was calculated using 2010 Osaka Census data [15]. Incidence was compared between sexes, using Fisher’s exact test. Considering the effect of endogenous female hormone on self-inflicted injuries, we divided eligible patients into the following two groups: the groups aged < =49 years or > =50 years according to the published data on mean age of menopause (49.5 ± 3.5 years) among Japanese females [16]. Incidence was also assessed between sexes by these two groups, using Fisher’s exact test.

Incidence of self-inflicted injuries based on temporal patterns (time of day, day of week, and season) was analyzed using Poisson regression models with risk ratios (RRs) and their 95 % confidence intervals (CIs). Time of day was divided into four 6-hour groups. For seasons, the period from April to June was defined as ‘spring: 1st quarter,’ July to September ‘summer: 2nd quarter,’ October to December ‘autumn: 3rd quarter’ and January to March ‘winter: 4th quarter’.

The incidence of self-inflicted deaths was also evaluated between the groups aged < =49 years or > =50 years by sex. We defined patients with hospital admission or death as severe self-inflicted injuries and the proportion of them was compared by sex using chi-square test. In the subgroup analysis, we also assessed self-inflicted deaths by sex and means. All statistical analyses were carried out using SPSS statistical package version 22.0 J (IBM Corp., Armonk, NY, USA). All tests were two-tailed, and *P*-values of <0.05 were considered statistically significant.

## Results

### Population

**Fig. 1:**
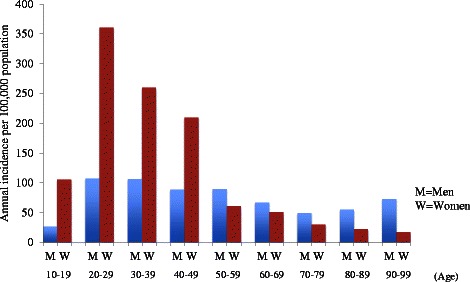
Annual incidence per 100,000 population in Osaka City of emergency patients with self-inflicted injuries by age group and sex

During the study period, a total of 633,359 emergency patients were documented in ambulance records in Osaka City. Among them, a total of 8,671 patients with 9,424 incidents of self-inflicted injuries were identified. Annual incidence of self-inflicted injuries per 100,000 men and women by 10-year strata age groups is shown in Fig. 1, respectively. Incidence was higher among women than men in the total population (136.9 and 82.6; *P* < 0.001). Among both sexes, self-inflicted injuries occurred from the 10–19 years, and the incidence reached its peak in 20–29 years (101.9 in men and 309.9 in women). In the group aged < =49 years, the incidence was higher among women than men (214.8 versus 93.3; *P* < 0.001), but it was inversely lower among women than men in the group aged > =50 years (49.0 versus 68.9; *P* < 0.001).

### Temporal patterns

**Fig. 2:**
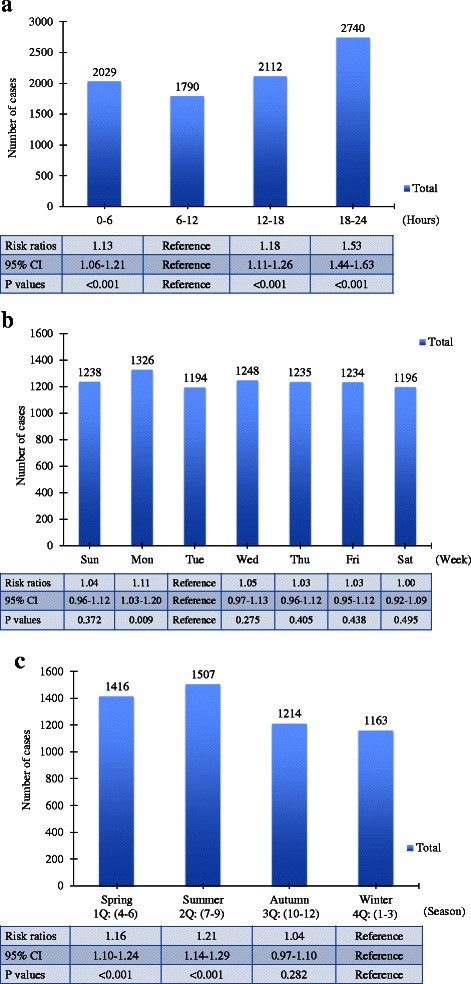
Number of emergency self-inflicted injury patients by temporal patterns such as **a** hour, **b** week, and **c** season. Tables below show RRs and their CIs by Poisson regression models

The number of self-inflicted injuries with temporal patterns is described in Fig. 2. The number by time of day was highest during the period of 18–24 h, which was 1.53-folds (95 % CI, 1.44-1.63) greater than the lowest period of 6–12 h (Fig. 2a). Regarding influence of day of week, self-inflicted injuries were most frequent on Mondays, least on Tuesdays (RR, 1.11; 95 % CI, 1.03-1.20 for Monday versus Tuesday) (Fig. 2b). As for seasons, 1.16-folds (95 % CI, 1.10-1.24) and 1.21-folds (95 % CI, 1.14-1.29) more cases were noted in spring and summer than in winter, respectively (Fig. 2c).

### Characteristics

Table 1 shows patient characteristics by their type of self-inflicted injuries. While 3340 (35.4 %) were reported from men, 6027 (64.0 %) were from women. Median age was 37 (interquartile 27–49) years. The types of self-inflicted injuries were as follows: 2965 (31.4 %) poisoning with sleeping pills or tranquillizers, 2190 (23.2 %) cutting and/or piercing wrist or arm, 1489 (15.8 %) hanging, 1436 (15.2 %) poisoning with other drug, 534 (5.7 %) jumping from heights, 491 (5.2 %) cutting and/or piercing other part of body, 121 (1.3 %) poisoning by other gas, 100 (1.1 %) drowning, and 98 (1.0 %) poisoning by CO. In this area, all CO poisoning were due to charcoal burning. There were no self-inflicted firearms in this study. Only 1493 (15.8 %) patients were transported to critical care medical centers and 4676 (49.6 %) to non-critical care medical centers, whereas the number of non-transported patients including cases with refusal of transport by themselves, only prehospital treatments at the scene, or death confirmed at the scene was 3255. Patients transported to medical institutions were treated at the following departments: 3086 (32.7 %) at internal medicine, 2889 (30.7 %) at general surgery, orthopedics, or neurosurgery, and 169 (1.8 %) at psychiatry.

**Table 1:** Characteristics of emergency self-inflicted injuries in Osaka City

	Population of Osaka City	Total		Poisoning sleeping pill or tranquilizer		Poisoning other drug		Poisoning by CO		Poisoning by other gas		Cutting and/or piercing wrist or arm		Cutting and/or piercing other part		Hanging		Jumping^c^		Drowning		*P* Values^¶^
		(*n* = 9424)		(*n* = 2965)		(*n* = 1436)		(*n* = 98)		(*n* = 121)		(*n* = 2190)		(*n* = 491)		(*n* = 1489)		(*n* = 534)		(*n* = 100)		
Men, *n*, (%)	1293798^d^	3340	(35.4)	766	(25.8)	381	(26.5)	64	(65.3)	72	(59.5)	513	(23.4)	242	(49.3)	1010	(67.8)	245	(45.9)	47	(47.0)	<0.001
Women, *n*, (%)	1371516^d^	6027	(64.0)	2192	(73.9)	1050	(73.1)	32	(32.7)	45	(37.2)	1674	(76.4)	249	(50.7)	451	(30.3)	281	(52.6)	53	(53.0)	
Unknown, *n*, (%)		57	(0.6)	7	(0.2)	5	(0.3)	2	(2.0)	4	(3.3)	3	(0.1)	0	(0.0)	28	(18.8)	8	(1.5)	0	(0.0)	
Age Category, year, *n*, (%)																						
10-19	216289	425	(4.5)	112	(3.8)	98	(6.8)	0	(0.0)	6	(5.0)	146	(6.7)	12	(2.4)	22	(1.5)	26	(4.9)	3	(3.0)	<0.001
20-29	337803	2425	(25.7)	831	(28.0)	450	(31.3)	14	(14.3)	32	(26.4)	702	(32.1)	99	(20.2)	165	(11.1)	115	(21.5)	17	(17.0)	
30-39	413633	2300	(24.4)	837	(28.2)	387	(26.9)	22	(22.4)	20	(16.5)	610	(27.9)	97	(19.8)	207	(13.9)	106	(19.9)	14	(14.0)	
40-49	362393	1819	(19.3)	661	(22.2)	266	(18.5)	29	(29.6)	22	(18.2)	391	(17.9)	108	(22.0)	227	(15.2)	95	(17.8)	20	(20.0)	
50-59	307103	854	(9.1)	248	(8.4)	101	(7.0)	13	(13.3)	9	(7.4)	159	(7.3)	55	(11.2)	208	(14.0)	51	(9.6)	10	(10.0)	
60-69	377586	671	(7.1)	145	(4.9)	65	(4.5)	9	(9.2)	16	(13.2)	101	(4.6)	65	(13.2)	195	(13.1)	49	(9.2)	26	(26.0)	
70-79	277163	392	(4.2)	65	(2.2)	36	(2.5)	0	(0.0)	1	(0.8)	45	(2.1)	37	(7.5)	161	(10.8)	38	(7.1)	9	(9.0)	
80-89	123535	127	(1.3)	19	(0.6)	11	(0.8)	0	(0.0)	3	(2.5)	16	(0.7)	15	(3.1)	55	(3.7)	8	(1.5)	0	(0.0)	
90-99	22186	19	(0.2)	0	(0.0)	0	(0.0)	0	(0.0)	0	(0.0)	8	(0.4)	0	(0.0)	9	(0.6)	2	(0.4)	0	(0.0)	
Unknown	23954	392	(4.2)	47	(1.6)	22	(1.5)	11	(11.2)	12	(9.9)	12	(0.5)	3	(0.6)	240	(16.1)	44	(8.2)	1	(1.0)	
Place, *n*, (%)																						
Home		7299	(77.5)	2653	(89.5)	1245	(86.7)	59	(60.2)	85	(70.2)	1814	(82.8)	372	(75.8)	1025	(68.8)	44	(8.2)	2	(2.0)	<0.001
Outside		1100	(11.7)	140	(4.7)	72	(5.0)	36	(36.7)	22	(18.2)	194	(8.9)	58	(11.8)	180	(12.1)	303	(56.7)	95	(95.0)	
Building		966	(10.3)	157	(5.3)	101	(7.0)	3	(3.1)	14	(11.6)	172	(7.9)	59	(12.0)	274	(18.4)	183	(34.3)	3	(3.0)	
Health care facility		59	(0.6)	15	(0.5)	18	(1.3)	0	(0.0)	0	(0.0)	10	(0.5)	2	(0.4)	10	(0.7)	4	(0.7)	0	(0.0)	
Call to the initiation of treatment by EMS, min, mean (SD)		7.0	(5.1)	6.9	(3.7)	6.9	(3.6)	8.0	(7.0)	11.4	(13.8)	6.5	(3.3)	6.8	(4.1)	6.9	(3.5)	6.4	(3.7)	15.8	(21.8)	<0.001
Call to hospital arrival, min, mean (SD)^a^		53.9	(31.1)	62.3	(31.7)	61.8	(31.0)	43.0	(21.1)	48.7	(25.4)	43.0	(20.6)	52.9	(33.0)	42.3	(32.0)	38.3	(21.7)	60.9	(32.8)	<0.001
Type of hospitals, *n* (%)																						
No transportation		3255	(34.5)	790	(26.6)	293	(20.4)	52	(53.1)	74	(61.2)	787	(35.9)	112	(22.8)	1031	(69.2)	92	(17.2)	24	(24.0)	<0.001
Non critical care medical center		4676	(49.6)	1871	(63.1)	877	(61.1)	13	(13.3)	18	(14.9)	1304	(59.5)	241	(49.1)	216	(14.5)	91	(17.0)	45	(45.0)	
Critical care medical center^b^		1493	(15.8)	304	(10.3)	266	(18.5)	33	(33.7)	29	(24.0)	99	(4.5)	138	(28.1)	242	(16.3)	351	(65.7)	31	(31.0)	
Type of transported department, *n* (%)^a^																						
Internal medicine		3086	(32.7)	1725	(58.2)	880	(61.3)	36	(36.7)	30	(24.8)	175	(8.0)	27	(5.5)	144	(9.7)	24	(4.2)	45	(45.0)	<0.001
General surgery, orthopedics, or neurosurgery		2889	(30.7)	385	(13.0)	229	(15.9)	10	(10.2)	17	(14.0)	1187	(54.2)	341	(69.5)	286	(19.2)	411	(78.6)	23	(23.0)	
Psychiatry		169	(1.8)	58	(2.0)	26	(1.8)	0	(0.0)	0	(0.0)	40	(1.8)	9	(1.8)	23	(1.5)	6	(0.1)	7	(7.0)	
Other		25	(0.3)	7	(0.2)	8	(0.6)	0	(0.0)	0	(0.0)	1	(0.6)	2	(0.4)	5	(0.3)	1	(0.0)	1	(1.0)	

*EMS* emergency medical service, *CO* carbon monoxide, *SD* standard deviation

^a^Calculated only for self-harms transported to institutions

^b^9.9 % (148/1493) were transported to critical care medical centers outside of Osaka City

^c^Among 534 patients, there were 3 patients with driving off the road and 36 patients with jumping in front of trains/other traffic

^d^Calculated for whole population in Osaka City

^¶^Comparison between the 9 groups were evaluated with Fisher exact test

### Outcomes

Table 2 shows patient outcomes by the type of self-inflicted injuries. The incidence of self-inflicted deaths among men was higher in the group aged > =50 years than in those aged < =49 years (128.5 versus 95.1; *P* < 0.001), but not among women (29.2 versus 32.9; *P* = 0.246). About one-fifth of patients (11.5 % refusal of transport by patients and 10.2 % only prehospital treatments at the scene) were not transported to hospitals. There were no significant sex differences in the proportion of transport refusal. Approximately one-third of patients who presented with self-inflicted injuries were only treated after transportation and were not admitted to a hospital. The proportion of hospital admissions was higher among women than among men (30.3 % [883/3340] vs. 26.4 % [1825/6027], *P* < 0.001). The proportion of self-inflicted deaths was overall 16.6 %, and was higher among men than among women (32.2 % [1075/3340] vs. 7.5 % [451/6027], *P* < 0.001). The most frequent type of self-inflicted deaths was hanging, which was responsible for 73.0 % (1142/1564) of all deaths. The proportion of severe self-inflicted injuries was greater among men than women (58.6 % [1958/3340] vs. 37.8 % [2276/6027], *P* < 0.001).

**Table 2:** Outcomes of emergency self-inflicted injuries in Osaka City

	Total		Sex					Poisoning sleeping pill or tranquilizer		Poisoning other drug		Poisoning by CO		Poisoning by other gas		Cutting and/or piercing wrist or arm		Cutting and/or piercing other part		Hanging		Jumping		Drowning		*P* Values†
			Men		Women		*P* Values*																			
	(*n* = 9424)		(*n* = 3340)		(*n* = 6027)			(*n* = 2965)		(*n* = 1436)		(*n* = 98)		(*n* = 121)		(*n* = 2190)		(*n* = 491)		(*n* = 1489)		(*n* = 534)		(*n* = 100)		
Refusal of transport by patients, *n*, (%)	1147	(11.5)	422	(11.8)	706	(11.2)	0.196	648	(21.9)	252	(17.5)	5	(5.1)	15	(12.4)	111	(4.3)	24	(4.3)	73	(4.6)	9	(1.7)	10	(10.0)	<0.001
Only prehospital treatments at the scene, *n*, (%)	965	(10.2)	242	(7.2)	723	(12.0)	<0.001	139	(4.7)	39	(2.7)	1	(1.0)	7	(5.8)	673	(30.7)	76	(15.5)	14	(0.9)	3	(0.6)	13	(13.0)	<0.001
No hospital admission after transportation, *n*, (%)	3040	(32.3)	718	(21.5)	2322	(38.5)	<0.001	1057	(35.6)	460	(32.0)	5	(5.1)	11	(9.1)	1185	(54.1)	192	(39.1)	67	(4.5)	35	(6.6)	28	(28.0)	<0.001
Hospital admission, *n*, (%)	2708	(28.0)	883	(26.4)	1825	(30.3)	<0.001	1113	(37.5)	681	(47.4)	31	(31.6)	28	(23.1)	217	(9.9)	179	(36.5)	193	(13.0)	233	(43.6)	33	(33.0)	<0.001
Death, *n*, (%)	1564	(16.6)	1075	(32.2)	451	(7.5)	<0.001	8	(0.3)	4	(0.3)	56	(57.1)	60	(49.6)	4	(0.2)	20	(4.1)	1142	(76.7)	254	(47.6)	16	(16.0)	<0.001
Death confirmed at the scene	1143	(12.1)	838	(25.1)	267	(4.4)		3	(0.1)	2	(0.1)	46	(46.9)	52	(43.0)	3	(0.1)	12	(2.4)	944	(63.4)	80	(15.0)	1	(1.0)	
Death confirmed at the hospital admission	421	(4.5)	237	(7.1)	184	(3.1)		5	(0.2)	2	(0.1)	10	(10.2)	8	(6.6)	1	(0.0)	8	(1.6)	198	(13.3)	174	(32.6)	15	(15.0)	

CO, carbon monoxide;

^*^Comparison between men and women were evaluated with chi-square test

^†^Comparison between the 9 groups (type of self-harms) were evaluated with Fisher exact test

As for the types of self-inflicted injuries, 37.5 % of patients with sleeping pill or tranquilizer poisoning, and 47.4 % of patients with some other drug poisoning were admitted to a hospital. Only 10.0 % of patients who cut and/or piercing their wrist or arm were admitted to a hospital, whereas 36.5 % of those who injured other parts of their bodies were admitted. The highest lethality rates of all the means were 76.7 % (1142/1489) of self-inflicted hanging. The percentage of self-inflicted deaths by jumping was 47.6 % (254/534). The proportion of self-inflicted deaths by poisoning by CO and poisoning by other gas was 57.1 % (56/98) and 49.6 % (60/121), respectively. The proportion of patients with death confirmed at the scene was 12.1 % (1143/9424) overall, and that confirmed at hospital admission was 4.5 % (421/9424).

Table 3 shows the lethality of self-inflicted injuries by sex and means. Men had a higher rate of death by self-inflicted hanging than women (82.8 % versus 62.3 %, *p* = 0.002).

**Table 3:** Lethality of self-inflicted injuries by sex and means

	Total*			Men			Women			*P* Values^†^
	Fatal	Total	% (Fatal/Total)	Fatal	Total	% (Fatal/Total)	Fatal	Total	% (Fatal/Total)	
Poisoning by sleeping pill or tranquilizer	8	2958	(0.3)	4	766	(0.5)	4	2192	(0.2)	0.217
Poisoning by Other drug	4	1431	(0.3)	3	381	(0.8)	1	1050	(0.1)	0.061
Poisoning by CO	54	96	(56.3)	36	64	(56.3)	18	32	(56.3)	1.000
Poisoning by Other gas	57	117	(48.7)	38	72	(52.8)	19	45	(42.2)	0.616
Cutting and/or piercing wrist or arm	4	2187	(0.2)	2	513	(0.4)	2	1674	(0.1)	0.237
Cutting and/or piercing other part	20	491	(4.1)	16	242	(6.6)	4	249	(1.6)	0.010
Hanging	1117	1461	(76.5)	836	1010	(82.8)	281	451	(62.3)	0.002
Jumping	246	526	(46.8)	126	245	(51.4)	120	281	(42.7)	0.246
Drowning	16	100	(16.0)	14	47	(29.8)	2	53	(3.8)	0.003

*CO* carbon monoxide

^*^Total = men + women

^†^The lethality was compared by sex

## Discussion

Using large-scale EMS records, we investigated characteristics and outcomes of emergency patients with self-inflicted injuries treated by EMS personnel. Our findings suggested the gender paradox that the proportion of self-inflicted deaths was higher among men than women, while the proportion of non-fatal self-inflicted injuries was higher among women than among men, particularly in the group aged < =49 years [17]. To our knowledge, this was one of the largest-scale studies assessing EMS-related self-inflicted injuries in Asia, and our findings showing the importance of the prevention for self-inflicted injuries as well as the gender paradox of self-inflicted injuries will provide important epidemiological information to improve psychiatric cares in prehospital emergency settings.

The present study demonstrated that the most frequent types of self-inflicted injuries were poisoning with drugs, followed by cutting and/or piercing skin and hanging. Our finding was similar to that in previous studies [6, 12, 18]. Hanging was the most common type of self-inflicted deaths, followed by jumping and poisoning by gas. The manner of self-inflicted deaths varied among countries. For example, firearms are most frequently used in the United States, as are pesticides in rural areas of developing countries [19, 20]. Self-inflicted deaths by CO poisoning from charcoal burning was common in East/Southeast Asia including Japan [20, 21]. In Japan, ever since charcoal burning suicide news made national headlines in 2003, the incidence has been increasing [20, 21]. This increase might be explained by the news of such charcoal burning suicide via the Internet or the widespread of Internet-related suicide pacts [20–23]. The most important factor for self-inflicted injuries and suicide is the accessibility to the means [24]. Therefore, in order to prevent self-inflicted injuries or suicide, there must be restriction of the access to the means.

As reported in a previous study from Australia, the present study underscored that self-inflicted injuries occur from a person’s 10–19 years, its incidence increased with age, and it reaches a peak at a person’s 20–29 years, and is higher among women than men in the group aged = <49 years [25]. Previous studies reported that the onset of self-inflicted injuries might be caused by puberty [26, 27]. The increasing trend of self-inflicted injuries during adolescence may be attributed to multiple factors as follows: economic status, educational status, abuse, bullying, negative life events such as parent death, environmental factors such as social transmission of self-inflicted injuries, and increasing prevalence of psychiatric disorders [28]. Social transmission of self-inflicted injuries or increasing prevalence of psychiatric disorders might have an especially strong influence on increasing the incidence of self-inflicted injuries among women [29, 30]. Although the effect of female hormones on self-inflicted injuries is still under debates [31], a report shows that female hormones may cause the increased prevalence of self-inflicted injuries among women at reproductive age (10–49 years) [32, 33]. Indeed, the higher incidence of self-inflicted injuries among women at reproductive age was also noted in this study. A previous study from Australia also reported that the increasing self-inflicted injuries trend seems to cease in a person’s latter 20–29 years [25]. Although it is not clear whether those previous findings may be plausible explanations for our findings, the time has come for us to take measures so that there is a prevention and intervention strategy for self-inflicted injuries among younger women.

In addition, the incidence of self-inflicted deaths among men aged > =50 years was higher than that in men aged = <49 years. Our findings here might be explained by psychiatric disorders, age-related disability, and social factors such as deaths of close relatives, loneliness from living alone, and socioeconomic status with increasing age [34, 35], but definite reasons for the higher incidence of self-inflicted deaths injuries among men were unclear. It is well-known that the incidence of severe self-inflicted injuries is higher among men than women regardless of age [1]. Indeed, the proportion of severe self-inflicted injuries was also greater among men than women in this study. A further detailed investigation is also needed to provide an effective suicide prevention strategy for men, especially elderly men.

Although there have been many studies on temporal patterns of suicide, little is known about those of self-inflicted injuries themselves [6, 12]. As shown in previous studies on self-inflicted injuries or suicide, the present study found that self-inflicted injuries were most frequent at 18–24 h or in the spring (1Q) and summer (2Q), and least frequent at 6–12 h or in winter (4Q), respectively [6, 36–38]. In Western countries, Monday is well-known as “blue Monday” in the field of psychiatry, when self-inflicted deaths occur most frequently [39]. The present study also showed that the highest incidence of self-inflicted injuries is on Monday. An epidemiological study on temporal patterns concerning self-inflicted injuries would be very important for the prevention and intervention regarding self-inflicted injuries or suicide, as would be hospital staffing and medical resource allocation as a health policy. Furthermore, these findings should be confirmed in other cohorts from different communities.

In Osaka City, Japan, many emergency patients who died from hanging and poisoning by CO were not transported to any hospitals. Importantly, these patients were not enrolled in the All-Japan Utstein registry of out-of-hospital cardiac arrests (OHCAs), because most of them had definite signs of death on EMS arrival [40]. Similarly, OHCA patients with rigor mortis, decapitation, incineration, dependent cyanosis, or decomposition were not transported. In other words, all resuscitated OHCA patients are transported to hospitals, but the prevalence of OHCAs in Japan based on the Utstein-style would underestimate them statistically, resulting in a high survival rate after OHCAs because of the exclusion of un-resuscitated patients. Therefore, establishing a comprehensive OHCA registry including OHCA patients who were not transported is needed to understand the actual situation regarding OHCAs in Japan.

The number of emergency patients with self-inflicted injuries is an urgent problem [1], and a comprehensive strategy will be of help in the prevention of self-inflicted injuries and suicide. Primary care providers, of course may well have an important role in preventing suicide. For example, a review reported that about twice as many patients who committed suicide had contact with primary care providers as mental health services within one month of their suicide [41]. Another recent systematic review also showed the effectiveness of intervention and prevention of suicide by using social network services [42]. Thus, to reduce emergency patients transported to hospitals by EMS, community-based interventions via primary care or the Internet may be of considerable help.

The present study has several limitations. First, in Japan, deceased patients who were not transported were managed by police, and their sex and age were not available in our ambulance records. Second, data used in the present study were based on ambulance records, and we did not find out the purpose/motivation of self-inflicted injuries such as suicidal intention, patients’ comorbidities, past history of suicide attempts or self-harms, detailed information about the type of drugs such as opioid or non-opioid drugs as well as legal or illegal drugs, or outcomes after hospital admission. However, we have been prospectively collecting such data on emergency patients in Osaka Prefecture since October, 2014 and will reveal more detailed information on emergency self-inflicted injuries in the future [43]. Third, although a previous study demonstrated that the number of patients who did not visit hospitals with self-inflicted injuries is about eight times as large as those who did, we did not, unfortunately, obtain any information on these patients [44], and we should, therefore, make a greater effort to grasp the total incidence of self-inflicted injuries in this area. Finally, our findings may not be generalizable to other districts because our study was conducted in a single big city.

From population-based ambulance records in Osaka City, the incidence of self-inflicted injuries treated by EMS personnel was higher in women than in men, but the incidence was more frequent in men than in women in the age group > =50 years old. The proportion of self-inflicted deaths was approximately one-sixth in whole self-inflicted injuries, and most frequent manner among deceased patients was by hanging, followed by jumping and poisoning by gas.

## Abbreviations

EMS: Emergency-medical-service
OECD: Organization for economic co-operation and development
WHO: World Health Organization
CO: Carbon monoxide
RR: Risk ratios
CI: Confidence interval
OHCA: Out-of-hospital cardiac arrest
SD: Standard deviation
